# Imaging genomic mapping of an invasive MRI phenotype predicts patient outcome and metabolic dysfunction: a TCGA glioma phenotype research group project

**DOI:** 10.1186/1755-8794-7-30

**Published:** 2014-06-02

**Authors:** Rivka R Colen, Mark Vangel, Jixin Wang, David A Gutman, Scott N Hwang, Max Wintermark, Rajan Jain, Manal Jilwan-Nicolas, James Y Chen, Prashant Raghavan, Chad A Holder, Daniel Rubin, Eric Huang, Justin Kirby, John Freymann, Carl C Jaffe, Adam Flanders, Pascal O Zinn

**Affiliations:** 1 Department of Diagnostic Radiology, M. D. Anderson Cancer Center, 1400 Pressler St; Unit 1482, Rm # FCT 16.5037, Houston, TX 77030, USA; 2 Massachussets General Hospital, Boston, MA, USA; 3 Department of Radiology, Emory University, Atlanta, GA, USA; 4 University of Virginia, Charlottesville, VA, USA; 5 Department of Radiology, New York University Medical Center, New York, NY, USA; 6 University of California San Diego Health System, San Diego, CA, USA; 7 Department of Radiology, San Diego Medical Center, San Diego, CA, USA; 8 Stanford University, Stanford, CA, USA; 9 Clinical Monitoring Research Program, Leidos Biomedical Research Inc., Frederick National Laboratory for Cancer Research, Frederick, MD 21702, USA; 10 NCI/NIH, Rockville, MD, USA; 11 Department of Radiology, Thomas Jefferson University Hospital, Philadelphia, PA, USA; 12 Department of Neurosurgery, Baylor College of Medicine, 1 Baylor Plaza, Houston, TX 77030, USA

**Keywords:** Radiogenomics, MRI segmentation, Glioblastoma, Imaging genomics, Invasion, Biomarker

## Abstract

**Background:**

Invasion of tumor cells into adjacent brain parenchyma is a major cause of treatment failure in glioblastoma. Furthermore, invasive tumors are shown to have a different genomic composition and metabolic abnormalities that allow for a more aggressive GBM phenotype and resistance to therapy. We thus seek to identify those genomic abnormalities associated with a highly aggressive and invasive GBM imaging-phenotype.

**Methods:**

We retrospectively identified 104 treatment-naïve glioblastoma patients from The Cancer Genome Atlas (TCGA) whom had gene expression profiles and corresponding MR imaging available in The Cancer Imaging Archive (TCIA). The standardized VASARI feature-set criteria were used for the qualitative visual assessments of invasion. Patients were assigned to classes based on the presence (Class A) or absence (Class B) of statistically significant invasion parameters to create an invasive imaging signature; imaging genomic analysis was subsequently performed using GenePattern Comparative Marker Selection module (Broad Institute).

**Results:**

Our results show that patients with a combination of deep white matter tracts and ependymal invasion (Class A) on imaging had a significant decrease in overall survival as compared to patients with absence of such invasive imaging features (Class B) (8.7 versus 18.6 months, p < 0.001). Mitochondrial dysfunction was the top canonical pathway associated with Class A gene expression signature. The *MYC* oncogene was predicted to be the top activation regulator in Class A.

**Conclusion:**

We demonstrate that MRI biomarker signatures can identify distinct GBM phenotypes associated with highly significant survival differences and specific molecular pathways. This study identifies mitochondrial dysfunction as the top canonical pathway in a very aggressive GBM phenotype. Thus, imaging-genomic analyses may prove invaluable in detecting novel targetable genomic pathways.

## Background

Recent advances in high throughput whole-genome glioblastoma (GBM) analyses have led to an increase in the understanding of gliomagenesis and elucidation of new molecular pathways [1,2]. However, the dismal prognosis of GBM remains largely unchanged with the median survival less than 2 years [3]. Heterogeneity in cellular composition, differential genomic expression profiles and therapy-resistant cancer stem cells within a single tumor (intra-tumor heterogeneity) and with the “same” tumor across different individuals (inter-individual heterogeneity) contribute largely to the lack of advancement in therapeutic approaches for these heterogeneous tumors and lie central to the failure of significant progress in the treatment of GBM [4,5]. Cellular invasion remains a significant cause of therapy failure as diffuse dissemination of tumor cells throughout the entire GBM brain including the normal appearing white matter precludes clean surgical margins. Moreover, glioma stem-like cells, thought to be involved in invasion, do not respond to current chemotherapeutic agents which target the active tumor core [6,7].

Magnetic resonance imaging (MRI) has been shown to non-invasively reflect the underlying tumor biological and pathological processes [7,8], tumor microenvironment [9], and genomic cancer composition [10-13]. Multiple preoperative imaging characteristics consistent with invasive tumor growth are documented [13,14]. Qualitatively, these include the 1) presence of either T1 contrast enhancement or increase T2/FLAIR hyperintensity involving the internal capsule, corpus callosum (unilateral, bilateral, or contralateral) or brainstem; 2) the presence of ependymal enhancement; and 3) the presence of pial enhancement [14]. Quantitatively, 3D volumetry of the peri-tumoral non-enhancing FLAIR hyperintensity has been validated to reflect an increase in gene signatures promoting cellular invasion and angiogenesis, as shown by our group previously [13]. In this current study, we sought to identify the invasive MRI characteristics in GBM and the implicated genes and microRNAs associated with qualitative invasive imaging signatures. Using our large-scale genomic database provided by The Cancer Genome Atlas (TCGA) and the imaging of the corresponding TCGA patients provided by The Cancer Imaging Archive (TCIA), we performed an MRI radiophenotype screen to identify key genes and molecular pathways associated with a very aggressive GBM radiophenotype.

## Methods

The collection of the original material and data of TCGA and TCIA study was conducted in compliance with all applicable laws, regulations and policies for the protection of human subjects, and any necessary approvals, authorizations, human subject assurances, informed consent documents, and IRB approvals were obtained [2].

### Patient population and TCGA and TCIA

We identified 104 (female 38: male 66; mean age 58 years; age range from 14 to 84 years) treatment-naïve GBM patients from TCGA whom had gene expression profiles and corresponding pretreatment MR imaging available in the TCIA. The TCGA is a National Cancer Institute (NCI) sponsored publicly available resource which has produced a multi-dimensional genomic and clinical data set of GBM and other cancers [2]. Image data used in this research were obtained from TCIA (http://cancerimagingarchive.net/) sponsored by the Cancer Imaging Program, DCTD/NCI/NIH [15]. The latter archive repository contains the imaging corresponding to the patients of the TCGA.

### Image acquisition and analysis

All images were downloaded from the NCI’s TCIA (http://cancerimagingarchive.net/) [15]. Image analysis was performed as previously published by our group [13,16]. Standard imaging parameters were used for each of the sequences as noted in the TCIA database [15].

### Image analysis

**Figure 1:**
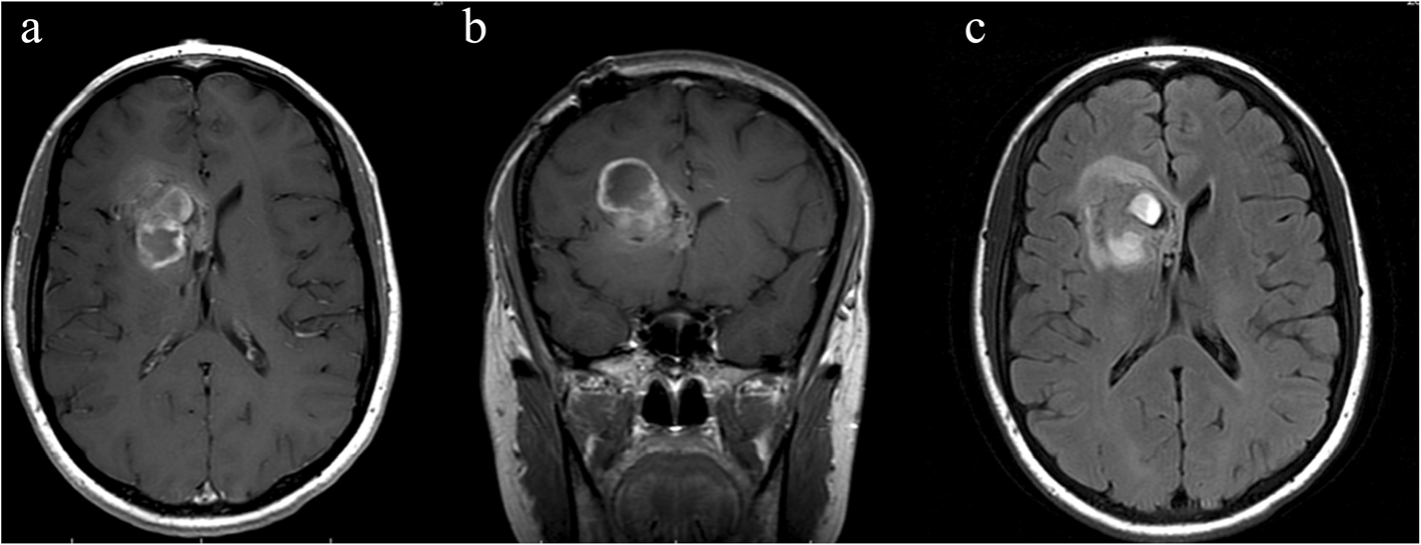
**Image example of qualitative invasive phenotype: ependymal extension.** 33 year old female patient with right frontal GBM. **(a)** Axial and **(b)** coronal post-contrast T1-weighted images demonstrate extension of enhancing tumor into the ependymal region of the frontal horn of the right lateral ventricle. **(c)** Axial FLAIR image demonstrates non-enhancing tumor as well to extend to the ependymal region.

For each patient, three board-certified neuroradiologists independently reviewed pre- and post- contrast axial T1-weighted MR images as well as axial T2-weighted FLAIR images. Images were analyzed using the Clearcanvas platform (ClearCanvas, Toronto, Canada; http://www.clearcanvas.ca/) and each physician entered specified imaging findings in an installed electronic case report form that implemented the VASARI feature set for human GBM [16-19]. Each board-certified neuroradiologist (C.H., 15 years of experience; A.F., 22 years; S.H., 5 years; M.W., 6 years; P.R., 4 years; R.R.C., 3 years; and M.J., 3 years) recorded a set of mark-ups for 30 imaging features describing the size, location, and morphology of the tumor.Included in the standardized VASARI feature-set criteria are qualitative visual assessments for key features of invasion: 1) deep white matter tract (DWMT) involvement [presence of either T1 contrast enhancement or increase T2/FLAIR hyperintensity involving the internal capsule, corpus callosum (unilateral, bilateral, or contralateral), or brainstem]; 2) the presence of ependymal enhancement; and 3) the presence of pial enhancement (Figure 1). Other parameters also analyzed were enhancing tumor crossing the midline, non-enhancing tumor crossing the midline, tumor extension into cortex, definition of the enhancing margin, definition of the non-enhancing tumor margin, proportion of edema, presence and absence of cysts and hemorrhage.

### Biostatistical image and survival analysis

Proportional hazards regression was performed with each of the following qualitative invasive covariates as a predictor of survival, taken individually: 1) DWMT involvement: presence of either T1 contrast enhancement or increase T2/FLAIR hyperintensity involving the internal capsule, corpus callosum (unilateral, bilateral, or contralateral), or brainstem; 2) ependymal enhancement; and 3) pial invasion. Proportional hazards regression was similarly performed on other imaging parameters as follows: enhancing tumor crossing the midline, non-enhancing tumor crossing the midline, tumor extension into cortex, definition of the enhancing margin, definition of the non-enhancing tumor margin, proportion of edema, presence and absence of cysts and hemorrhage. Bonferroni correction was performed. Kaplan-Meier method (log-rank test) was used to compute overall median survival. Data table preparation was done in Microsoft Excel 2010 and then loaded into the JMP Pro 9.01 (SAS Institute, Cary NC) and R-project (Bioconductor platform) software packages for statistical analysis.

Patients were subsequently classified based on presence or absence of the invasion MRI phenotypes that were statistically significant. Class A patients demonstrated the presence of all the independent statistically significant parameters of invasion. Patients who had no invasion phenotypes or presence of only one were assigned to Class B.

### Biostatistical image- genomic analysis

Affymetrix level 1 mRNA and Agilent level 1 microRNA data were downloaded from the public TCGA data portal (April 2013) (http://cancergenome.nih.gov/). Level 1 mRNA Affymetrix CEL file analysis was performed in R project, a free statistical computing platform, (http://www.r-project.org/) using the Bioconductor platform (http://www.bioconductor.org/). Robust Multi-Array (RMA) algorithm was used for normalization [20].

In each patient, a total of 13,628 genes (22,267 hybridization probes) were analyzed for significance and differential fold regulation in Class A versus Class B groups by Comparative Marker Selection (CMS) (Broad Institute, MIT, Cambridge, MA, http://www.broadinstitute.org/cancer/software/genepattern/). CMS is a statistical method that uses permutation testing to identify differentially regulated genomic events in one versus another predefined patient group [21].

The top 100 most positively and the top 100 most negatively correlated mRNAs in the Class A group versus the Class B group were then analyzed with Ingenuity Pathway Analysis (IPA) (http://www.ingenuity.com). IPA provides insight into the molecular and chemical interactions, cellular phenotypes, and disease processes of a predefined set of genes and microRNAs. By means of IPA, our top molecular targets for the Class A group were analyzed in a comprehensive way and thus pertinent canonical pathways and functional networks accounting for an invasion radiophenotype were uncovered. Upstream transcriptional factor analysis was done using IPA.

## Results

### Univariate proportional hazards regression model fit for 11 potential predictors of survival

Univariate proportional hazards regression models with each of the 11 predictors of survival was performed. Of these 11 invasive imaging predictors, 3 imaging biomarkers were statistically significant. [Unadjusted p-values: Ependymal (EP) involvement, P = 0.005; deep white matter tract (DWMT) involvement {presence of either T1 contrast enhancement or increase T2/FLAIR hyperintensity involving the internal capsule, corpus callosum (unilateral, bilateral, or contralateral), or brainstem}, P = 0.002; enhancement across the midline, P = 0.0003]. Bonferroni adjustment was then performed. [Adjusted p-values: Ependymal (EP) involvement, P = 0.058; Hazard Ratio (HR) =1.812, Concordance = 0.593, 95% CI (1.19-2.77); deep white matter tract (DWMT) involvement {presence of either T1 contrast enhancement or increase T2/FLAIR hyperintensity involving the internal capsule, corpus callosum (unilateral, bilateral, or contralateral) or brainstem}, p = 0.027; HR =1.926, Concordance = 0.6, 95% CI (1.25-2.96); enhancement across the midline, P = 0.003; Concordance = 0.546, HR =3.48, 95% CI (1.70-7.15)]. We fit a final proportional hazards regression model including these three predictors together. All of the regression coefficients were statistically significant [P = 0.033, HR = 1.616, 95% CI (1.0-2.5); P = 0.015, HR = 1.738, 95% CI (1.1-2.7); and P = 0.038, HR = 2.219, 95% CI (1.0-4.7), for ependymal involvement, deep white matter tract involvement and enhancement across the midline, respectively]. This analysis was performed using the 'survival' library [22] in the R statistics package (http://www.R-project.org).

### Invasive imaging phenotypes

Deep white matter tract (DWMT) involvement: Enhancement across the midline and presence of either T1 contrast enhancement or increase T2/FLAIR hyperintensity involving the internal capsule, corpus callosum (unilateral, bilateral, or contralateral), or brainstem

**Figure 2:**
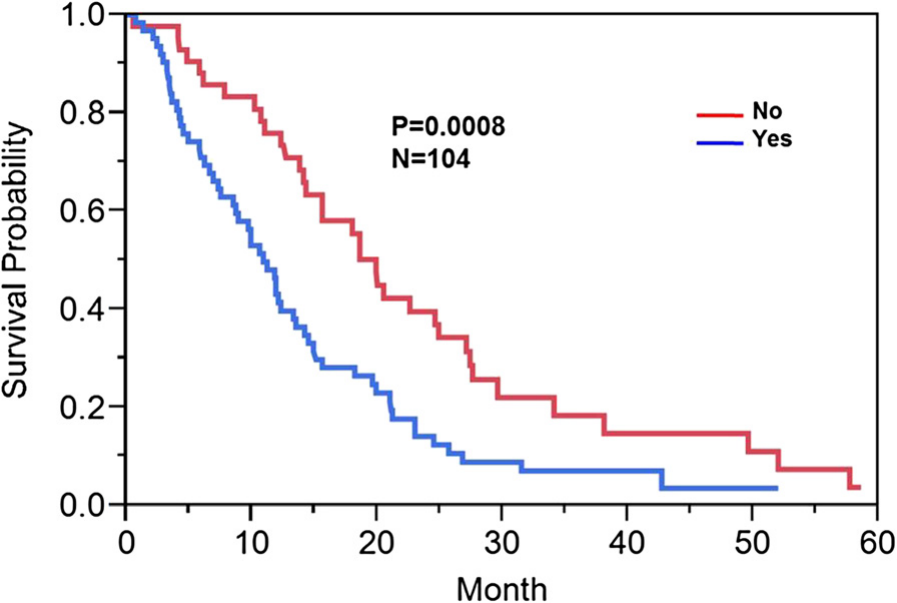
**Kaplan Meier survival curve: Deep white matter tract [DWMT] involvement.** Kaplan Meier method was used to compute overall median survival. Those who had involvement versus no involvement of the DWMT demonstrated a median overall survival of 10.9 months versus 19.9 months (p < 0.0008).

**Figure 3:**
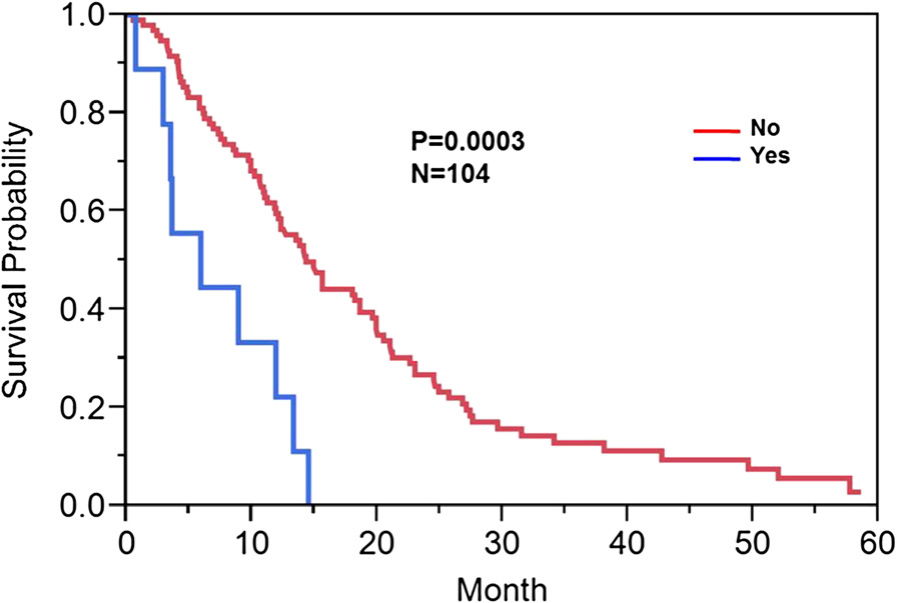
**Kaplan Meier survival curve: enhancing tumor across midline/corpus callosum.** Kaplan Meier method was used to compute overall median survival. Those who had enhancement across the midline versus those patients with absence of enhancement across the midline demonstrated a median overall survival of 9 months versus 14.3 months (p < 0.0003).

Forty percent (42/104) of patients had involvement of the internal capsule, corpus callosum (unilateral, bilateral, or contralateral), or brainstem either by T1 contrast enhancement or increase T2/FLAIR hyperintensity. Sixty percent (62/104) of patients had no involvement of these structures. Twenty-six percent (9/35) demonstrated enhancement across the midline (across the corpus callosum). Ninety-one percent (95/104) of patients did not demonstrate enhancing tumor across the midline/corpus callosum.Those who had involvement of the DWMT demonstrated a median overall survival of 10.9 months (95% CI: 7.5-13.3 months) versus 19.9 months (95% CI: 14.1-24.9 months) (p < 0.0008) (Figure 2). Of those who had enhancement across the midline, there was a statistically significant difference in overall survival [5.9 (95% CI: 0.7-13.3 months) versus 14.3 months (95% CI: 11.9-18.6 months) (p < 0.0003)] in those patients with presence versus absence of enhancement across the corpus callosum (Figure 3).

### Ependymal (EP) enhancement

**Figure 4:**
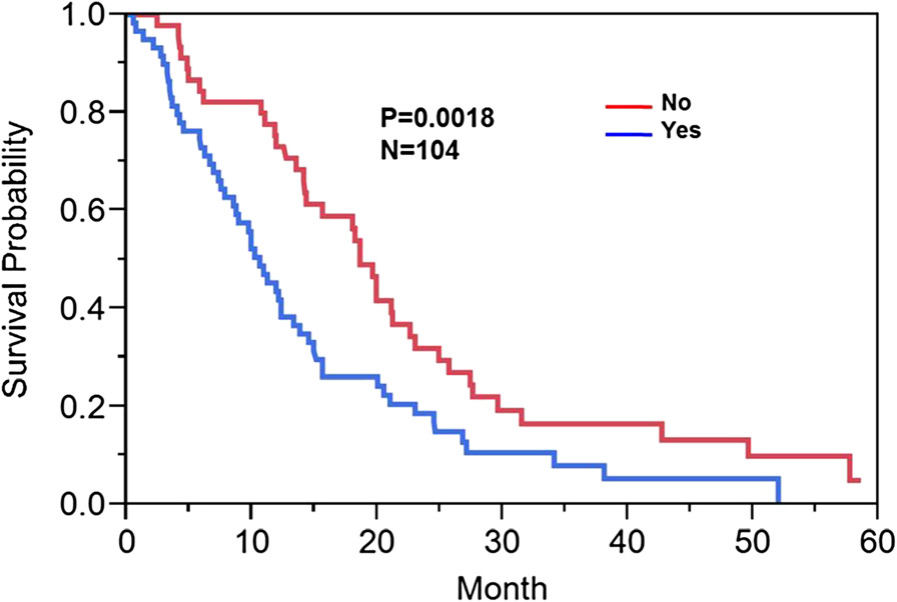
**Kaplan Meier survival curve: Ependymal [EP] involvement.** Kaplan Meier method was used to compute overall median survival. Those who had ependymal tumor involvement versus no ependymal tumor involvement demonstrated an overall survival of 10.6 versus 18.6 months (p = 0.0018).

Forty- three percent (45/104) of patient did not demonstrate extension of enhancing or non-enhancing tumor to the ependymal region. Fifty-seven percent (59/104) of patients had ependymal involvement by tumor. Involvement of the ependymal region was associated with a decrease in overall survival of 10.6 (95% CI: 7.8-13.3 months) versus 18.6 (95% CI: 14.2-22.6 months) months (p = 0.0018) (Figure 4).

### Invasion classes and genomic signatures

**Figure 5:**
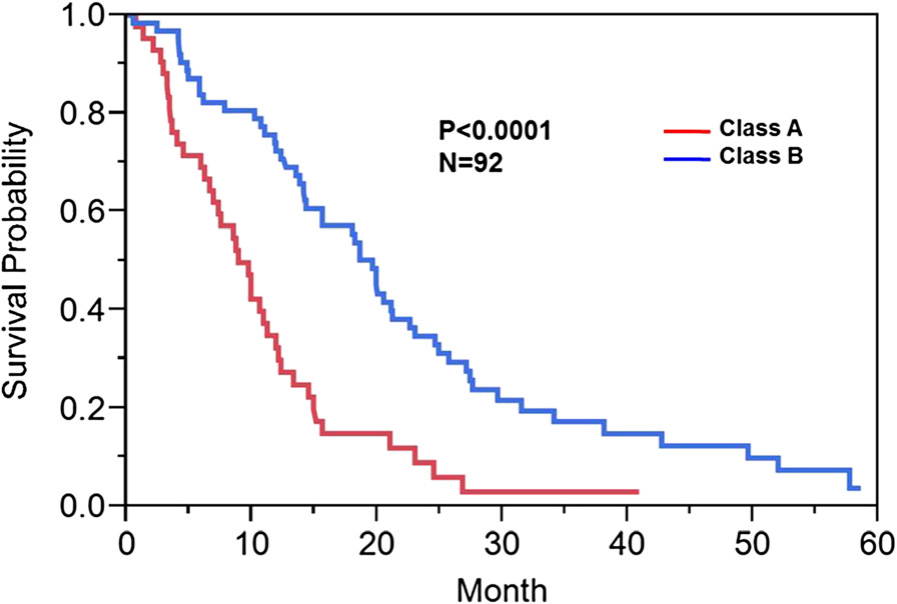
**Kaplan Meier survival curve: Class A versus Class B.** Kaplan Meier method was used to compute overall median survival. Those patients in Class A (invasive phenotypes) versus those patients in Class B (without invasive features or only one invasive feature) demonstrated an overall survival of 8.7 versus 18.6 months (p<0.001).

**Figure 6:**
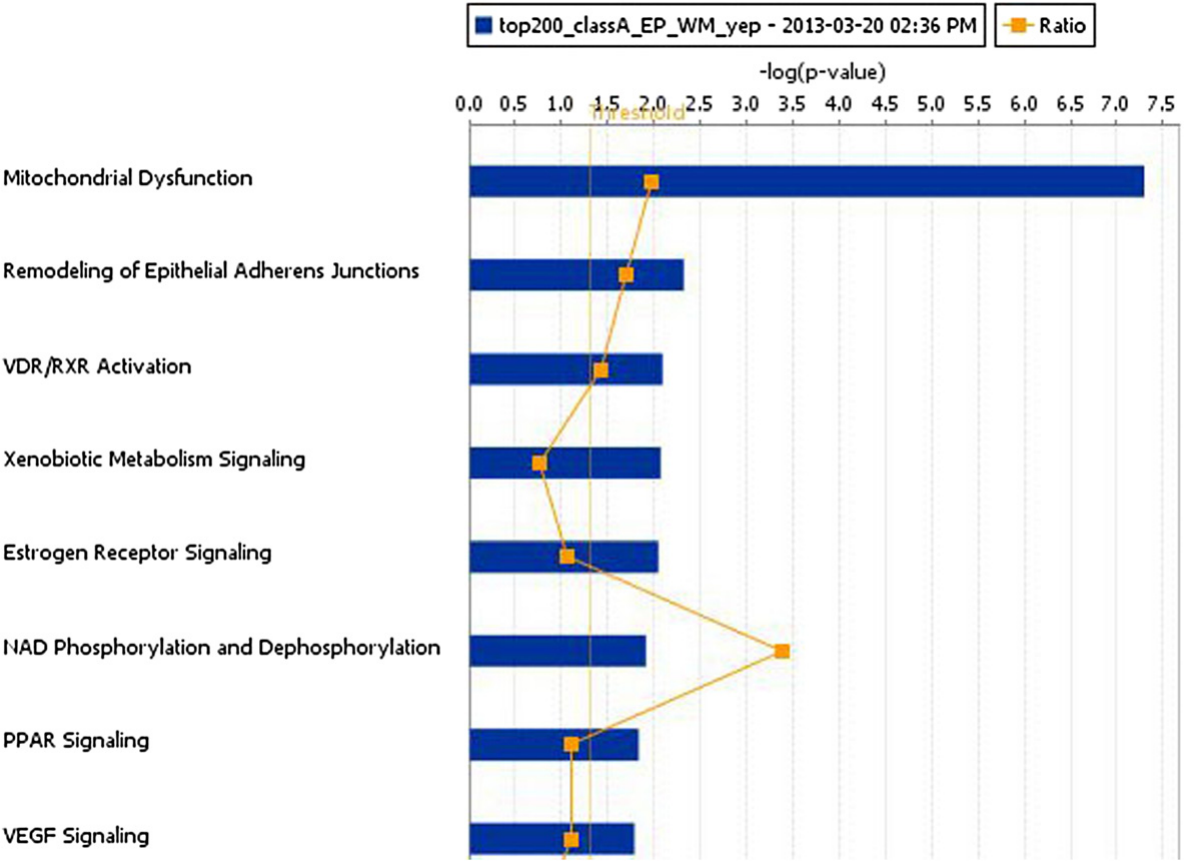
**Canonical pathways associated with Class A patients.** Canonical pathway analysis was performed using IPA. The top canonical pathway was mitochondrial dysfunction in Class A patients with invasive phenotypes.

**Figure 7:**
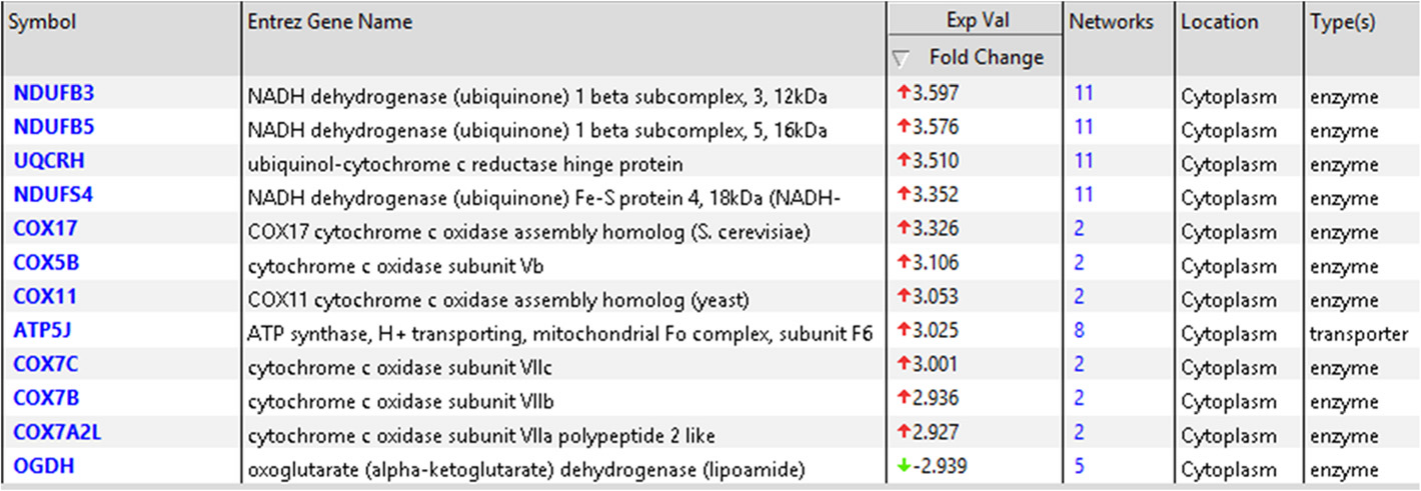
**The molecules associated with the mitochondrial dysfunctional canonical pathway.** In the fold change column, highly up-regulated genes (red color) and a single down-regulated gene (green color) were presented.

**Figure 8:**
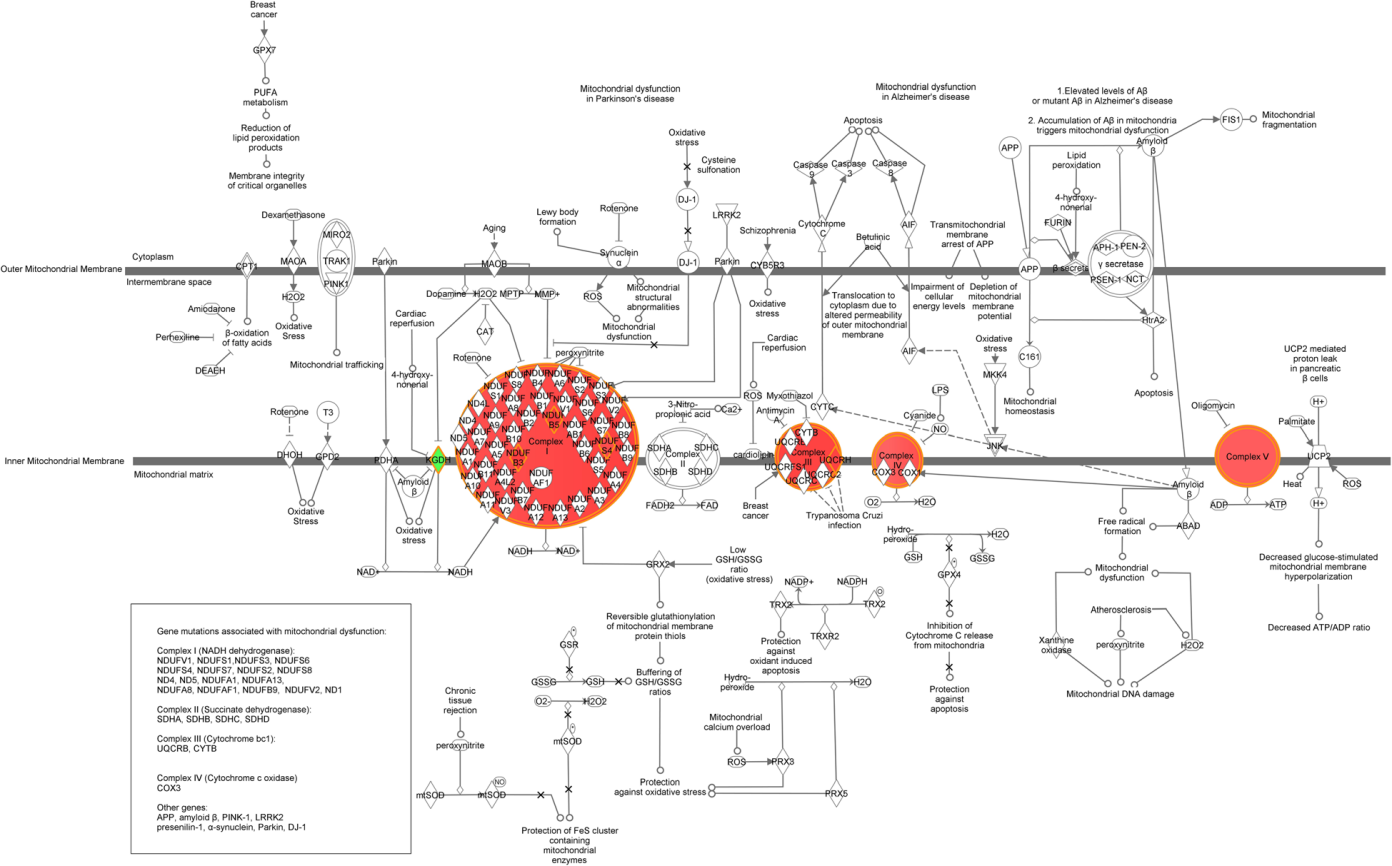
**Mitochondrial pathway demonstrating location of dysfunctional molecules along its spectrum.** Up-regulated genes were labeled with red color and down-regulated gene was labeled with green color.

**Figure 9:**
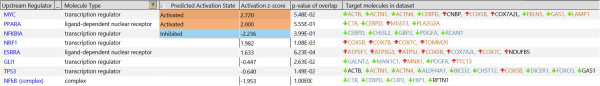
**Transcriptional factor analysis.** Transcriptional factor analysis was performed using IPA to predict the potential transcription factors involved and their activation or inhibition states in Class A versus Class B groups. MYC and PPARA were predicted to be activated and NFKB1A was predicted to be inhibited.

A total of 92 patients had comprehensive clinical and genomic annotation for creation of imaging genomic signatures. Given that the DWMT and EP imaging radiophenotypes were significantly prognostic (p = 0.015 and p = 0.033) in the proportional hazards model, we used these two invasion parameters to develop our invasion imaging classes. The imaging parameter of tumor across the midline was disregarded for further analysis since it only contained 9 patients that were also present in the DWMT and EP groups. Patients with both ependymal and deep white matter tract involvement tumor spread were then grouped into a single class (Class A; invasive phenotype group) and those without involvement or involvement of only one of those parameters were assigned to a second class (Class B). The patients with involvement of both variables demonstrated a statistically significant worse prognosis (8.9 months) than either variable alone (EP alone = 20 months; DWMT alone = 18.9 months) or no infiltration at all (18.6 months) which demonstrated similar prognosis. Forty percent (36/92) of patients were categorized into Class A and 60% (56/92) of patients into Class B. Class A demonstrated a decrease in survival when compared to Class B [8.9 (95% CI: 6.6-11.2 months) versus 19.6 (95% CI: 14.2-22.6 months) months, p < 0.001)] (Figure 5). A proportional hazard ratio demonstrated that the classification was a stronger predictor of survival (p = 0.0003) than tumor size (p = 0.35) and patient age (p = 0.93) (data not shown). The top canonical pathway associated with Class A (invasive phenotype group) was mitochondrial dysfunction (p = 2.59E-08) (Figures 6, 7 and 8). Transcription Factor Analysis (Figure 9) demonstrated *MYC* oncogene activation and inhibition of NF-*K*B inhibitor-alpha (NFKBIA) in class A.

## Discussion

In this study, we demonstrate that those patients with specific invasive imaging signatures have a highly significant decrease in overall survival and an associated distinctive genomic expression signature. MR imaging has been shown to predict patient survival and correlate with underlying genomic events in GBM [12,13,16]. Specifically, the ependymal involvement, deep white matter tract involvement and enhancement across the midline were statistically significant in predicting overall survival; and *MYC* oncogene activation and inhibition of NF-*K*B inhibitor-alpha (NFKBIA) were seen in class A. These findings are concordant with those published in the literature [14]. Furthermore, the location of tumor invasion was more important than the size of the tumor in predicting survival (p = 0.0003 versus p = 0.35).

The invasive imaging biomarker signature classes, Class A (invasive phenotype) and Class B, demonstrated a significant 9.9 months (8.7 versus 18.6 months, p < 0.0001, Figure 5) survival disadvantage in Class A. Class A patients reflected tumor compositions which have genes involved in invasion and oxidative stress/mitochondrial biology. In patients in Class A, the top canonical pathway was mitochondrial dysfunction (Figures 6 and 7). Furthermore, our classification screen demonstrated activation of *MYC* by upstream regulator analysis. The results of mitochondrial dysfunction and upstream transcription factor *MYC* activation are consistent with the finding that *MYC* can activate the transcription of target genes that increase mitochondrial biogenesis [23,24]. The findings of altered metabolism in our GBM patients is concordant with the literature [25,26]. Our patients with an invasive imaging signature demonstrated worse survival and altered metabolism. Altered metabolism is one of the important hallmarks of tumor cells [27,28]. Most cancer cells rely on aerobic glycolysis, a phenomenon termed “the Warburg effect” [29], the most well-known metabolic abnormality in cancer cells. This is unlike that of normal cells which rely on mitochondrial oxidative phosphorylation to generate the energy needed for cellular processes. In the Warburg effect [29], cancer cells have defects in the mitochondria processes and there is an increased glycolysis with lactate secretion and mitochondrial respiration even in the presence of oxygen [30]. This was also seen in our Class A patients (Figures 6, 7 and 8). Furthermore, many of the metabolism genes whose mutations can cause cancers are mitochondrial genes [31-33]. Energy metabolism associated genes *IDH1* and *IDH2* mutations are seen in secondary GBM [34,35]. The *P53* tumor suppressor and *MYC* oncogene are well-documented master regulators of metabolism [36]. The activation of *MYC* facilitates the Warburg effect (aerobic glycolysis) and induces glycolysis and glutaminolysis, two typical metabolism alterations present in cancer cells [37]. It has been well known that *MYC* target genes are involved in the maintenance of stem cell self-renewal ability and tumorigenesis [38,39]. Furthermore, aberrant activation of *MYC* expression in cancers provide sufficient energy and anabolic substrates for uncontrolled cell growth and proliferation in the context of the tumor microenvironment [36]. Thus, *MYC*-mediated altered cancer cell energy metabolism can be a potential target for the development of new anticancer therapies. Thus, the literature supports our findings which demonstrated activation of *MYC* in those patients with a high invasion phenotype (Class A).

**Figure 10:**
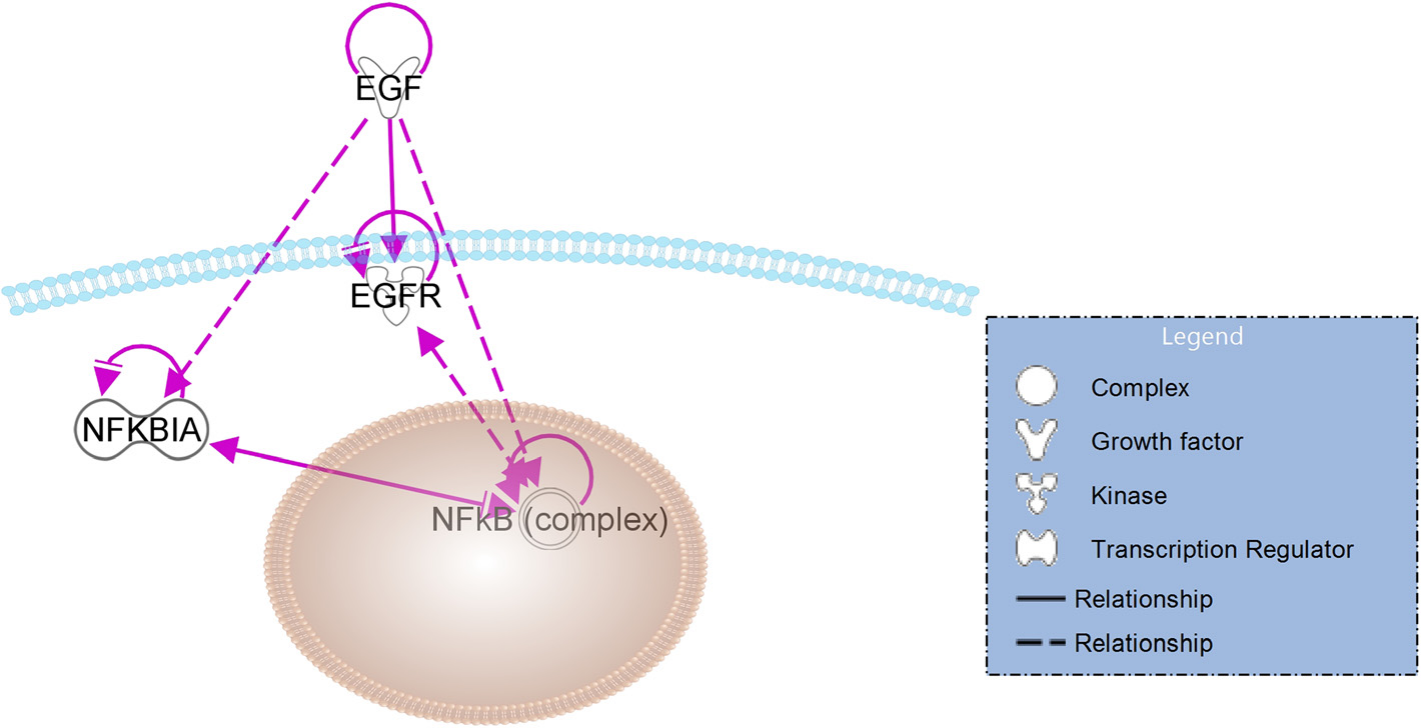
**Link between NFKBIA and EGFR pathway in Glioblastoma.** The relationship between EGFR and NF-kB signaling was performed using IPA Path Designer tool. Solid line indicates direct biological relationship. Dashed line indicates indirect biological relationship. The relationship between molecules were supported by IPA knowledge base. EGF signaling to NF-kB is affected by NFKBIA inhibition.

In our study, we found that NFKBIA was predicted to be the top inhibited transcriptional factor in Class A by transcription factor analysis. Nuclear factor of *k*-light polypeptide gene enhancer in B-cells (NF-*k*B) is a transcription factor that is activated by the epidermal growth factor receptor (EGFR) pathway [40] and shows aberrant constitutive activation in GBM [41,42]. NFKBIA represses the NF-*k*B and thus signaling from the EGFR and NF-*k*B pathways [43]. The finding of NFKBIA inhibition in this study suggests an interesting link between NF-*k*B pathway and EGFR signaling in GBM (Figure 10). Mutations in NFKBIA have been described in multiple cancers including GBM cell lines suggesting its function as a tumor suppressor [44]. Thus, its predicted inhibition is associated with increased oncogenicity and resistance to therapy, primarily mediated by its anti-apoptotic activity [41,45]. This data supports our genomic transcription factor signature identified by MRI; those patients in Class A demonstrated a poor survival when compared to Class B (8.7 versus 18.6 months, p < 0.0001) and had inhibition of the NFKBIA transcription factor. NF-*k*B inhibitors have been shown to induce cell death in GBM [46] and support the possibility of NF-*k*B as a potential target for cell death induction for GBM therapy. The development of therapeutics targeted against the NF-*k*B pathway can possibly address the chemo-resistance seen in GBM therapy today.

In summary, our study demonstrates for the first time imaging features that uncover and predict metabolic and mitochondrial dysfunction in GBM. The effect of the tumor microenvironment on cancer and the Warburg effect is increasingly being recognized as important modulators in genetics and epigenetics as well as facilitators of cancer development [2]. It is now believed that a better understanding of the mechanisms of the Warburg effect and altered metabolism may ultimately lead to more effective treatments for GBM and cancer, in general. This study shows that the proposed classification based on invasive GBM imaging biomarker signatures yields significant differences in survival and distinct genomic signatures that identify mitochondrial dysfunction as a possible driver for very aggressive GBM phenotypes and resistance to therapy. Certain limitations exist and are currently being addressed in new prospective clinical trials and novel animal model experiments. The known inherent limitation of the TCGA data is that surgical samples obtained for genomic analysis were not done using image-guidance and thus the location of biopsy is unknown. Given tumor heterogeneity in GBM, tissue sampling under image-guidance is needed to obtain more accurate specimens. Currently, we are performing a prospective image-guided biopsy study at our institution. Further, *in-vitro* and *in-vivo* testing in animal models is also being done in our lab for subsequent validation of our findings and to identify potential therapeutic targets for GBM treatment.

## Conclusion

Patients with specific invasive imaging signatures of ependymal involvement, deep white matter tract involvement and tumor extension across the midline have a highly significant decrease in overall survival and an associated distinctive genomic expression signature. *MYC* oncogene activation and inhibition of NFKBIA was seen in class A. This is a significant finding as these pathways can have a possible role as a target of therapeutic intervention.

## Abbreviations

GBM: Glioblastoma; TCGA: The cancer genome atlas; TCIA: The cancer imaging archive; VASARI: Visually accessible rembrandt images; MRI: Magnetic resonance imaging; IRB: Institutional review board; NCI: National cancer institute; DCTD: Division of cancer treatment and diagnosis; NIH: National institute of health; DWMT: Deep white matter tract involvement; FLAIR: Fluid attenuated inversion recovery; CMS: Comparative marker selection; IPA: Ingenuity pathway analysis; EP: Ependymal.

## Competing interests

The authors declare that they have no competing interests.

## Authors’ contributions

RRC and POZ designed and coordinated the overall study. RRC, MV, JW and POZ conducted the imaging genomics, bioinformatics and biostatistics analysis. RRC, DAG, SNH, MW, RJ, MNJ, JYC, PR, CAD and AF were involved in the image analysis. DR, EH, JK, JF, CCJ and AF collected and developed pipeline for the imaging data for TCIA. All authors read and approved the final manuscript.

## Pre-publication history

The pre-publication history for this paper can be accessed here:

http://www.biomedcentral.com/1755-8794/7/30/prepub
